# Dr. David Dale Owen

**Published:** 1861-01

**Authors:** 


Dr. David Dale Owen, who had earned an enviable reputation as a student of geology and the kindred sciences, died on the thirteenth of November. At the time of his decease, Dr. Owen was State Geologist of Indiana.

